# Identifying immune cell infiltration and effective diagnostic biomarkers in Crohn’s disease by bioinformatics analysis

**DOI:** 10.3389/fimmu.2023.1162473

**Published:** 2023-08-09

**Authors:** Rong Huang, Wenjia Wang, Ziyi Chen, Jing Chai, Qin Qi, Handan Zheng, Bingli Chen, Huangan Wu, Huirong Liu

**Affiliations:** ^1^ Key Laboratory of Acupuncture and Immunological Effects, Yueyang Hospital of Integrated Traditional Chinese and Western Medicine, Shanghai University of Traditional Chinese Medicine, Shanghai, China; ^2^ Shanghai Research Institute of Acupuncture and Meridian, Shanghai University of Traditional Chinese Medicine, Shanghai, China

**Keywords:** Crohn’s disease, fibrosis, ssGSEA, immune cells, biomarker

## Abstract

**Background:**

Crohn’s disease (CD) has an increasing incidence and prevalence worldwide. It is currently believed that both the onset and progression of the disease are closely related to immune system imbalance and the infiltration of immune cells. The aim of this study was to investigate the molecular immune mechanisms associated with CD and its fibrosis through bioinformatics analysis.

**Methods:**

Three datasets from the Gene Expression Omnibus data base (GEO) were downloaded for data analysis and validation. Single sample gene enrichment analysis (ssGSEA) was used to evaluate the infiltration of immune cells in CD samples. Immune cell types with significant differences were identified by Wilcoxon test and Least Absolute Shrinkage and Selection Operator (LASSO) regression analysis. Differentially expressed genes (DEGs) were screened and then subjected to Gene Ontology (GO) and Kyoto Encyclopedia of Genes and Genomes (KEGG) functional correlation analysis, as well as protein-protein interaction (PPI) network analysis. The cytoHubba program and the GSE75214 dataset were used to screen for hub genes and plot Receiver operating characteristic (ROC)curves to screen for possible biomarkers of CD based on diagnostic efficacy. The hub genes of CD were correlated with five significantly different immune cells. In addition, validation was performed by real time quantitative PCR (RT-qPCR) experiments in colonic tissue of CD intestinal fibrosis rats to further identify hub genes that are more related to CD intestinal fibrosis.

**Results:**

The DEGs were analyzed separately by 10 algorithms and narrowed down to 9 DEGs after taking the intersection. 4 hub genes were further screened by the GSE75214 validation set, namely COL1A1, CXCL10, MMP2 and FGF2. COL1A1 has the highest specificity and sensitivity for the diagnosis of CD and is considered to have the potential to diagnose CD. Five immune cells with significant differences were screened between CD and health controls (HC). Through the correlation analysis between five kinds of immune cells and four biomarkers, it was found that CXCL10 was positively correlated with activated dendritic cells, effector memory CD8^+^ T cells. MMP2 was positively correlated with activated dendritic cells, gamma delta T cells (γδ T) and mast cells. MMP2 and COL1A1 were significantly increased in colon tissue of CD fibrosis rats.

**Conclusion:**

MMP2, COL1A1, CXCL10 and FGF2 can be used as hub genes for CD. Among them, COL1A1 can be used as a biomarker for the diagnosis of CD. MMP2 and CXCL10 may be involved in the development and progression of CD by regulating activated dendritic cell, effector memory CD8^+^ T cell, γδ T cell and mast cell. In addition, MMP2 and COL1A1 may be more closely related to CD intestinal fibrosis.

## Introduction

Crohn’s disease (CD) belongs to inflammatory bowel disease (IBD), which is an autoimmune disease. The prevalence of CD in western countries is more than 0.3%, and the incidence rate in newly industrialized country is increasing (1, 2). CD, as a heterogeneous disease with multiple confounding factors, is related to genetic and environmental factors, dysbiosis of the microbiota, imbalance of innate and adaptive immune responses, and dysfunction of the epithelial intestinal barrier (3, 4). Among them, both innate immunity and adaptive immunity play important roles in the occurrence and development of CD. Therefore, exploring biomarkers with diagnostic significance in CD and revealing the immune mechanism of CD are the key to understanding and treating CD, and are also the focus of current research.

Studies have found that the mucosal immune system of patients with CD is over activated and abnormal cytokine response occurs. For example, IL-17 and IL-23 are related to the pathogenesis of CD. IL-23 belongs to the IL-12 cytokine family and can enhance and affect the expansion of pathogenic T helper type 17 (Th17) through various mechanisms. Macrophages are considered to be the main source of IL-23 in the intestine, and can form crosstalk with T cell subpopulations and innate lymphocytes in the intestine, thereby promoting intestinal inflammatory response (5). CD patients have defects in the number and inhibitory function of regulatory T cells (6). In addition, other immune cells, such as dendritic cells (DC) and Mast cell, also play important roles in the pathogenesis of CD (7, 8). At present, most studies are carried out from one or a class of immune cells and Longitudinal study are carried out, but immune cells do not play a role alone. Therefore, we urgently need a systematic method to evaluate the role of immune cells in CD, and further explore the key genes related to immune cells.

**Figure 1:**
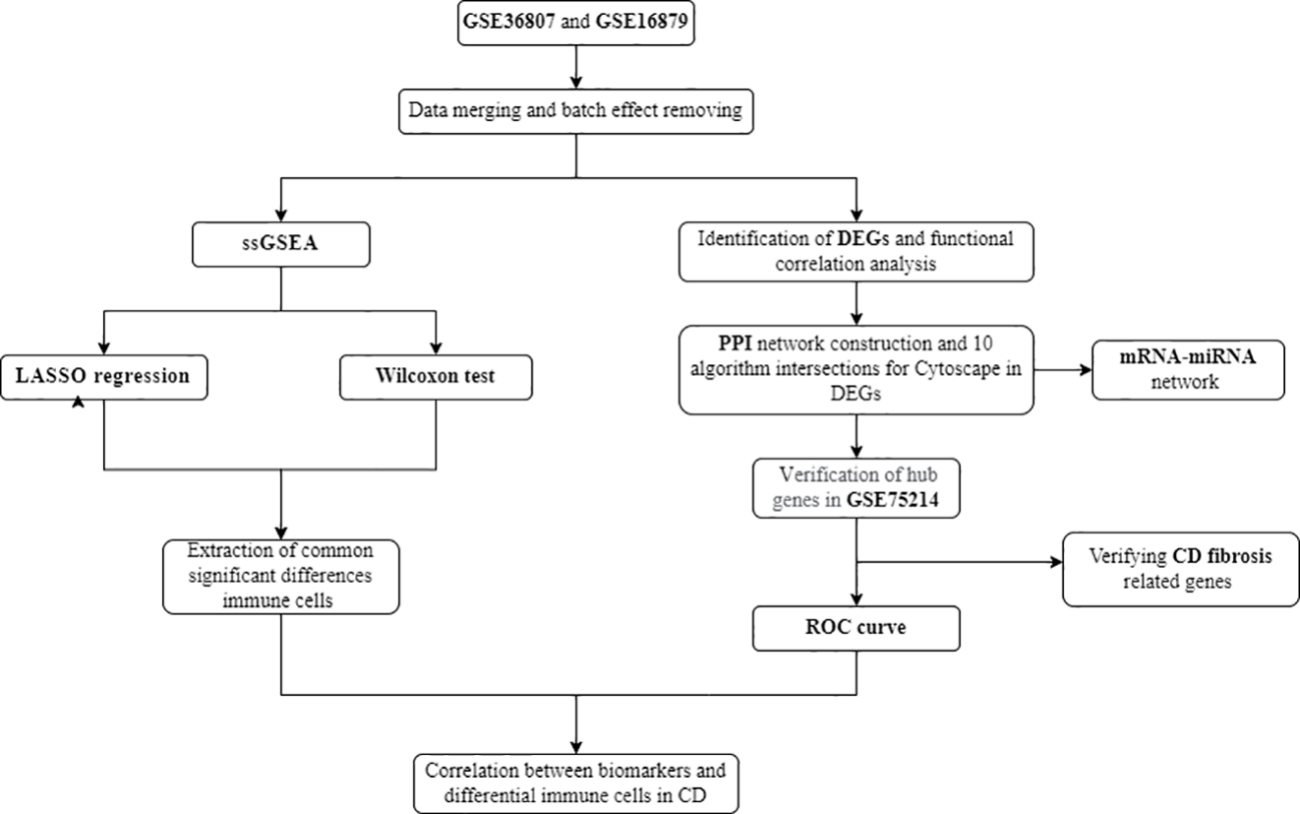
Flow chart of the analysis process conducted in this study.

As one of the most common complications of CD, intestinal fibrosis (IFB) can lead to intestinal stenosis and bowel obstruction. It is currently believed that CD intestinal fibrosis involves pathological processes such as fibroblast migration and proliferation, epithelial mesenchymal transition (EMT), extracellular matrix deposition (ECM), which affect the structure and function of intestinal tissue and lead to irreversible organ damage (9, 10). Within 10 years after CD diagnosis, 70% of patients will develop fibrotic stenosis (11, 12). However, at present, CD fibrosis is mostly diagnosed through invasive pathological examination, and due to diagnostic difficulties, early intervention is not possible. The disease often develops to an irreversible stage, and even becomes important during inevitable surgery. Research has found that inflammation is an important driving factor in inducing intestinal fibrosis, but when intestinal inflammation is effectively controlled, the process of intestinal fibrosis cannot be completely blocked. Simply controlling inflammation can only slow down the pathological process of fibrosis, but cannot prevent its continued development (11, 13). Therefore, as one of the important complications of CD, intestinal fibrosis in Crohn’s disease remains a key issue that needs to be addressed in clinical practice. It is particularly important to pay attention to and conduct in-depth research on the mechanisms of intestinal fibrosis in CD, and to find important molecular targets. The analysis process is shown in **Figure 1**.

## Materials and methods

### Data source

Microarray data of intestinal mucosal tissues from CD and HC meeting the criteria were downloaded from the GEO database (http://www.ncbi.nlm.nih.gov/geo/). The selection criteria included (1) mRNA expression profiles by microarray; (2) each dataset contained both intestinal mucosa from CD patients and normal intestinal tissues from healthy people; (3) each dataset contained ≥20 samples; (4) subjects were ≥18 years old. The GPL570 platform datasets GSE36807 and GSE16879 were finally selected as the test set, and the GPL6244 platform dataset GSE75214 was selected as the validation set. A total of 13 CD patients were included in the GSE36807 dataset, including 5 active stage patients and 8 remission stage patients (disease activity is determined based on symptoms such as abdominal pain and defecation, combined with extracolon manifestations, the presence of abdominal masses, blood inflammatory markers, and colon biopsy results). The specific Montreal L type data of Crohn’s disease are shown in **Table 1**. The samples of CD patients did not specify whether there were ulcers at the biopsy site; Seven HC individuals with normal colon were screened for colon cancer. All 20 samples were from colon tissue. The biopsy site for CD patients in the GSE16879 dataset is a location that is at a certain distance from the ulcer but with visible inflammatory manifestations; 12 HC patients were selected as individuals with normal intestinal mucosa for polyp screening, with 6 samples from colon tissue and 6 samples from ileum tissue; Out of 37 CD samples, 19 were from colon tissue and 18 were from ileum tissue. The GSE75214 dataset defines the visible ulcer under endoscopy as the active phase, but does not provide a specific location for biopsy; Among the CD patient samples, 67 were from ileum tissue, 8 were from colon tissue, and 22 HC patients were screened for polyps with normal intestinal mucosa. Among them, 11 samples were from colon tissue and 11 samples were from ileum tissue (**Table 1**).

**Table 1:** Basic information of selected datasets.

Characteristics	GSE36807	GSE16879	GSE75214
**Platform**	GPL570	GPL570	GPL6244
**Sample source**	Biopsy	Biopsy	Biopsy
**HC (n=41)**	7	12	22
**Ileum**	–	6	11
**Colon**	7	6	11
**CD (n=125)**	13	37	75
**Ileum**	1	9	67
**Active**	–	9	51
**Inactive**	–	–	16
**Colon**	6	14	8
**Active**	–	14	8
**Inactive**	–	–	–
**Ileocolon**	4	14	–
**Active**		14	
**Inactive**		–	
**Colon+EGD**	1	–	–
**Not confirmed**	1	–	–
**Attribute**	Test	Test	Verification
**Author/References**	Montero Melendez T (14)	Arijs I (15)	Jabara HH (16)

EGD, esophagogastroduodenoscopy.

### Data preprocessing

Download the raw and series matrix files of GSE36807 and GSE16879. For the raw data, the probe expression matrix was extracted and normalized using the R package (version 3.6.3) “affy”. The probe expression matrices were transformed into gene expression matrices using the platform annotation file. For a gene corresponding to multiple probes, the average value was taken as the expression value of the gene. Probes that correspond to multiple molecules are removed. The filtered data were removed from the two data sets using the “ComBat” function of the R package “sva”, and UMAP analysis was performed using the R package “umap”. The results were visualized by the R package “ggplot2”.

### Immune cell infiltration analysis

Immune cell infiltration between CD and normal tissues was explored by single sample gene enrichment analysis (ssGSEA). The Wilcoxon test and Least Absolute Shrinkage and Selection Operator (LASSO) regression analysis were used to screen out 15 and 14 immune cells with significant differences between the two groups, respectively(*p*<0.05), and the intersection of the two retained five immune cells as the final significantly different immune infiltrating cells. The Wilcoxon test uses wilcox.test in R, and the lasso regression uses the “glmnet” package. “corplot”, “vioplot”, “ggplot2” and “glment “ are used to visualize the results.

### Screening for differential genes and functional enrichment analysis

Differentially expressed genes (DEGs) were analyzed for CD and healthy controls using the R package “limma”. The thresholds of |log2FC|>1 and *p*.adj< 0.05 were used to screen DEGs. The results were visualized by heat map and volcano map with the R package “ComplexHeatmap” and “ggplot2”.Functional enrichment analysis was performed by converting gene IDs with the R package “org.Hs.eg.db” and analyzing DEGs GO and KEGG with the R package “clusterProfiler”. Under the threshold of *p*.adj<0.05 and qvalue<0.2, the functional and signaling pathways with significant enrichment were screened. The results of the enrichment analysis were visualized by the R package “ggplot2”.

### PPI network construction, screening and validation of Hub genes

PPI networks were constructed based on all DEGs using the online analysis tool STRING (https://string-db.org), setting higher filtering conditions (score>0.7). The downloaded interaction information files were imported into Cytoscape (v3.9.1) using the first 10 algorithms of cytoHubba, namely MCC (Maximum Clique Centrality), DMNC (Density of Maximum Neighborhood Component), MNC (Maximum Neighborhood Component), Degree, EPC (Edge Percolated Component), BottleNeckEcCentricity, Closeness, Radiality and Betweenness (BC), the top 60 genes of each algorithm were selected and the genes were screened by applying the R package “UpSet”. The key gene clusters were screened by MCODE (Molecular Complex Detection), and the screening conditions were set to degree cutoff=2, node score cutoff=0.2, k-core=2, max depth=100, and the top three scoring gene networks were selected for display. Through further validation of the dataset GSE75214, more reliable hub genes were screened.

### Prediction of targeting miRNAs

Four online miRNA databases, Targetscan (v8.0), miRWalk (v3.0), miRTarBase (v8.0) and miRDB (v6.0), were used to predict targeting miRNAs for nine genes. miRNAs that appeared in at least three databases were selected, and based on the targeting relationships of these genes with miRNAs, visualize and analyze the co-expression network in CytoScape.

### Analyzing the diagnostic validity of biomarkers

In the GSE75214 dataset, logistic models were constructed using the R package “glm” function, “pROC” for ROC analysis, and “ggplot2 “ for visualization. The area under the curve (AUC) was used to predict the diagnostic validity of CD biomarkers.

### Correlation analysis between hub gunes and immune cells

Using Spearman’s rank correlation analysis in R software, explore the correlation levels between four hub genes and five immune cells that significantly increase in CD, and visualize them using R package “ggplot2”.

### Quantitative real-time PCR

Total RNA was extracted from rat colon tissue using the over-column method according to the manufacturer’s instructions. RNA reverse transcription was performed using the color reverse transcription kit (EZBioscience). Quantitative PCR was performed using 2×color SYBR Green qPCR Master Mix (EZBioscience) in Roche LightCyclerTM 480. Standardization was performed using β-actin. The amplification and melting curves were confirmed at the end of the reaction and the data were calculated using the 2^-ΔΔCt^ method (**Table 2**).

**Table 2:** Gene sequences and primers were designed as follows.

NM	Primer name	Series (5’-3’)
NC_051345.1	Rat COL1A1 Forward primer	GCTTGAAGACCTATGTGGGTATAA
	Rat COL1A1 Reverse primer	GGGTGGAGAAAGGAACAGAAA
NM_019305.2	RatFGF2 Forward primer	AGCGGCTCTACTGCAAGAAC
	Rat FGF2 Reverse primer	TGGAGCTGTAGTTTGACGTGT
NM_031054.2	Rat MMP2 Forward primer	GCCCAGAGACTGCTATGTCC
	Rat MMP2 Reverse primer	GCTGGTGCAGCTCTCATACT
NM_139089.2	Rat CXCL10 Forward Primer Rat CXCL10 Reverse Primer	CGGTGAGCCAAAGAAGGTCTA CTAGCCGCACACTGGGTAAA

## Results

### Immune infiltration ssGSEA analysis

**Figure 2:**
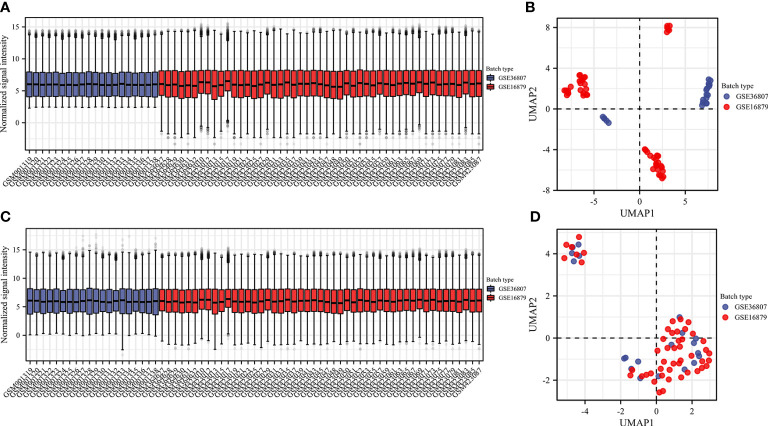
Data preprocessing. Box plot and uniform manifold approximation and projection were performed to remove batch correction of GSE36807 and GSE16879. **(A, B)** before batch correction and **(C, D)** after batch correction.

**Figure 3:**
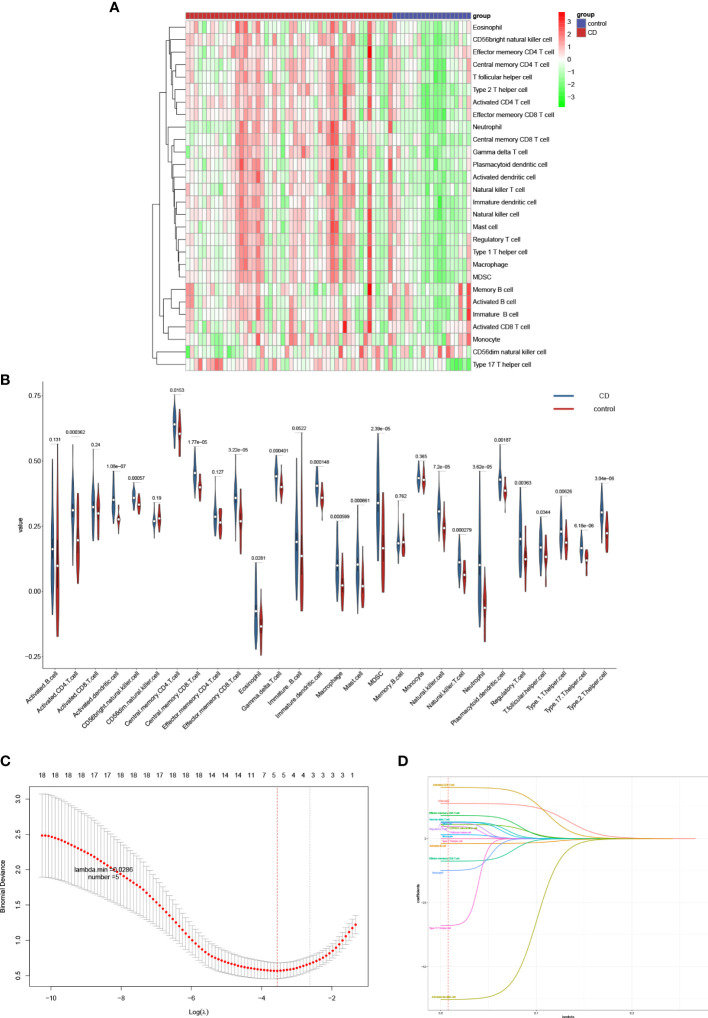
Analyzing immune cell infiltration and identifying the significantly different infiltrates of immune cells in CD and normal tissues. The composition of 28 species of immune cells in each sample was showed in a heatmap **(A)**. **(B)** Wilcoxon test and **(C, D)** LASSO regression were used to analyze the different infiltrates of immune cells.

We selected two microarray datasets GSE36807 and GSE16879 from the GPL570 platform, including a total of 50 CD samples and 19 HC samples. From before batch correction (**Figures 2A, B**) and after batch correction (**Figures 2C, D**), it can be seen that the differences between batches were removed and the data were well normalized after processing. First, the correlation with 22 immune cells in each sample is given in the form of a heat map (**Figure 3A**). Two different algorithms, Wilcoxon test and LASSO regression, were used to distinguish significant differences in immune cell infiltration in CD and HC. The results of the Wilcoxon test were shown in the violin plot (**Figure 3B**), which presented 21 immune cells with *p*<0.05; the LASSO regression results (**Figures 3C, D**) showed that five immune cells were included. The five immune cells common to both methods were extracted, namely effector memory CD8^+^ T cells, neutrophils, mast cells, activated dendritic cells, and γδ T cells.

### Screening for differential genes and associated functional analysis

**Figure 4:**
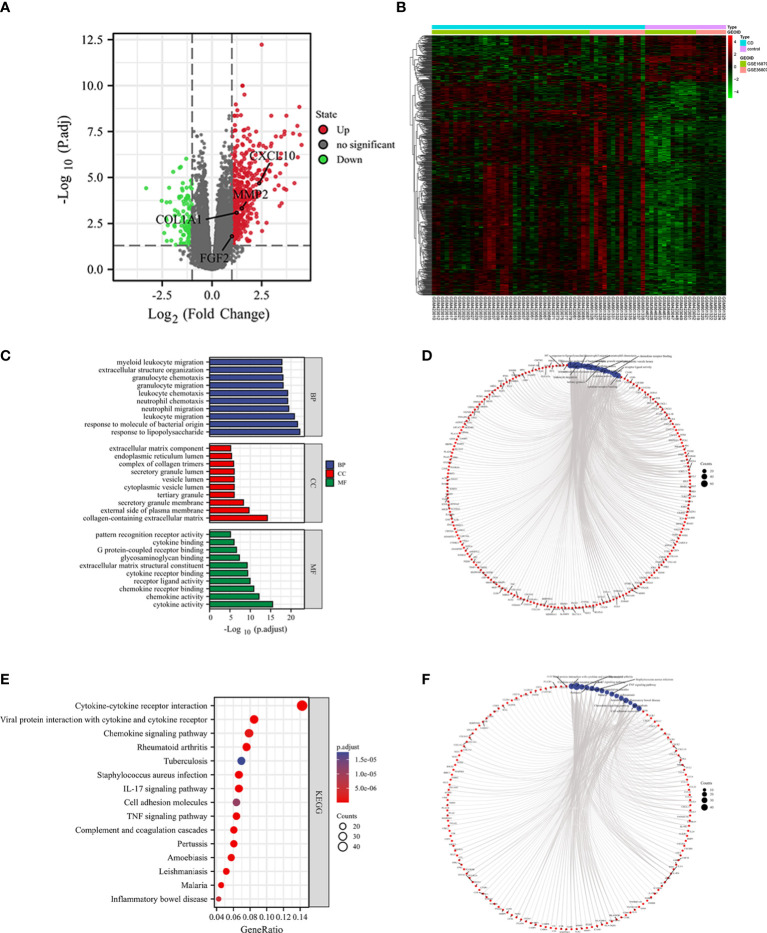
Identification of DEGs and functional correlation analysis by GO and KEGG. DEGs were visualized by volcano map **(A)** and heat map **(B)**. The results of GO were presented using bar plot **(C)** and circle graph **(D)**. The results of KEGG were showed by bubble plot **(E)** and circle graph **(F)**.

A total of 634 differential genes were identified in CD compared to HC samples, containing 521 up-regulated genes and 113 down-regulated genes. The differential genes were visualized using heat map and volcano map (**Figures 4A, B**). GO term was used to analyze the functional enrichment of DEGs involved in biological process (BP) closely related to the immune response. It involves the chemotactic and migratory responses of leukocytes, again dominated by neutrophils. As well as the cellular response to lipopolysaccharide (LPS), a component of the outer wall of the cell wall of bacteria, Gram-negative bacteria. The molecular functions (MF) involved are mainly related to cytokine activity, chemokine activity, receptor ligand activity, etc. The top 10 entries were selected according to the threshold *p*.adj<0.05 to draw a bar graph, and the top 5 entries for each of BP, CC (cellular component), and MF were selected to draw a circle graph (**Figures 4C, D**). Combined with the results of KEGG pathway enrichment showed that DEGs were closely related to immune response-related signaling pathways. They were mainly enriched in signaling pathways such as cytokine-cytokine receptor interaction, interleukin-17, tumor necrosis factor and inflammatory bowel disease. The top 10 pathways of KEGG were selected according to the threshold *p*.adj<0.05 to draw bubble diagrams, and the top 15 pathways were drawn as circles (**Figures 4E, F** ).

### Identification and validation of Hub genes

**Figure 5:**
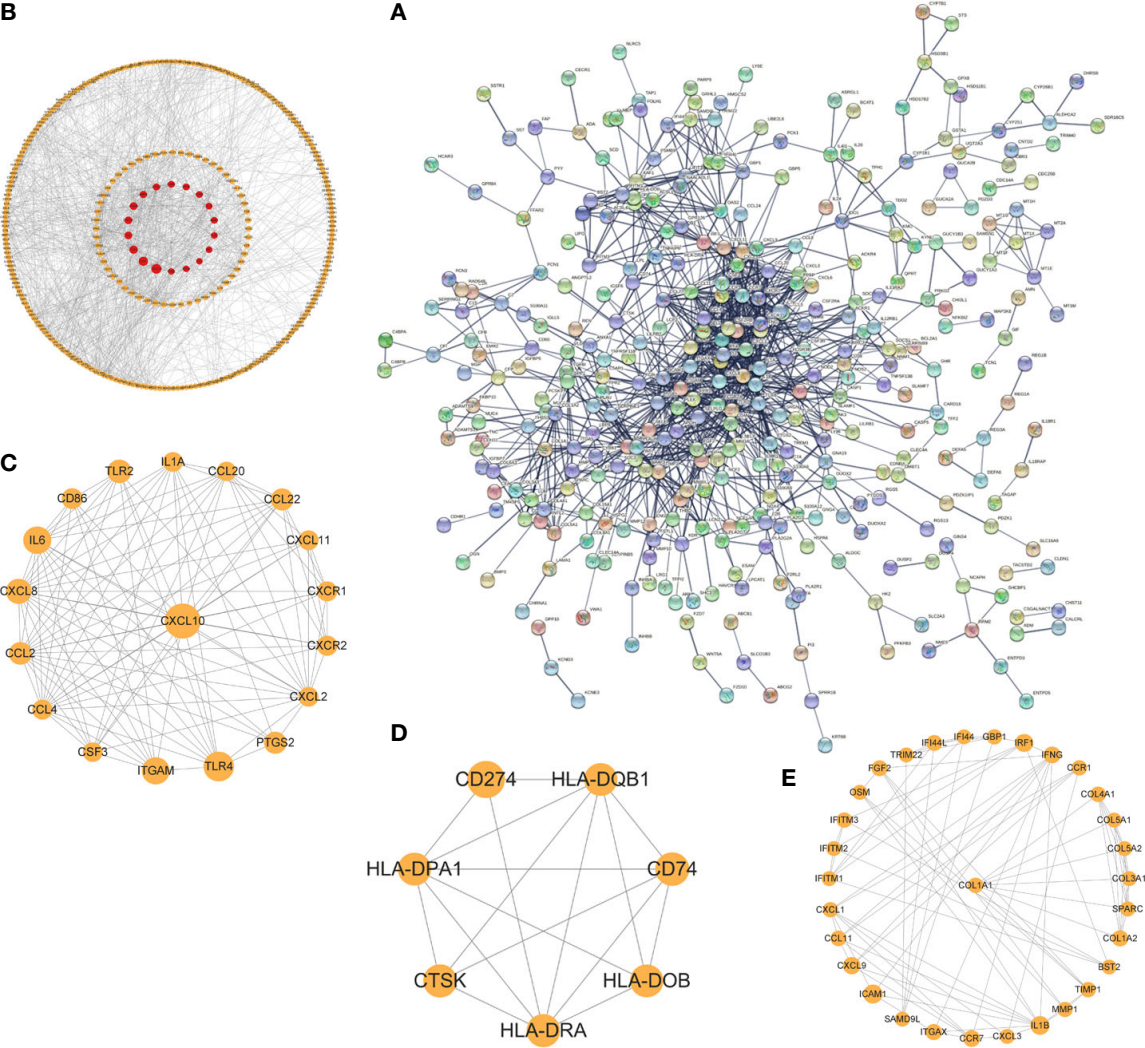
The PPI network and cluster modules. PPI network of DEGs was conducted by STRING software **(A)**. Visualization of PPI networks of DEGs using Cytoscape software **(B)** and screening of the 3 most important gene cluster modules by MCODE program **(C–E)**.

**Figure 6:**
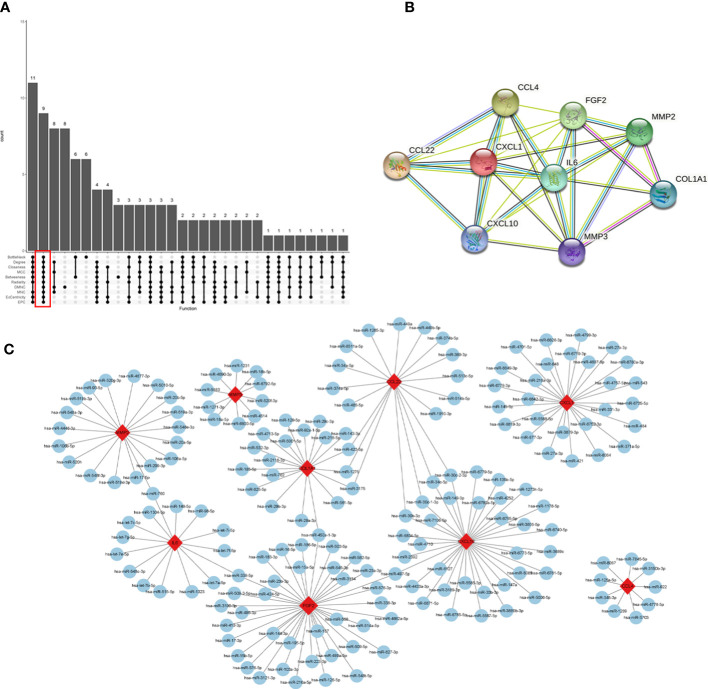
Identification of hub genes and a co-expressed network of mRNAs and target miRNAs. Hub genes were screened by the R package “Upset” **(A)**. Visualization the degree of interaction of 9 hub genes by STRING software **(B)**. The mRNA-miRNA co-expressed network was constructed by Cytoscape including 181 nodes and 192 edges **(C)**.

The interaction network (PPI) between proteins encoded by DEGs was constructed by STRING, which contained 590 nodes and 1177 edges with an average node degree value of 3.99, and visualized after hiding the protein nodes that were not connected (**Figure 5A**). The data were imported into Cytoscape and visualized after setting the node size by BC value (**Figure 5B**). Then MCODE was applied to filter the three gene cluster modules with scores >5, cluster 1 (score 12.118, containing 18 nodes and 103 edges), cluster 2 (score 5.667, containing 7 nodes and 17 edges), and cluster 3 (score 5.600, containing 31 nodes and 84 edges) (**Figures 5C–E**). Next, the top 60 genes from each of the 10 algorithms were filtered with the R package “UpSet” to identify 9 DEGs (**Figure 6A**), namely IL6, CXCL10, CCL22, CXCL1, CCL4, FGF2, MMP3, MMP2, COL1A1. It was visualized through the PPI network (**Figure 6B**). In addition, an mRNA and miRNA co-expression network consisting of 9 genes, 181 nodes and 192 edges was mapped using Cytoscape (**Figure 6C**).

**Figure 7:**
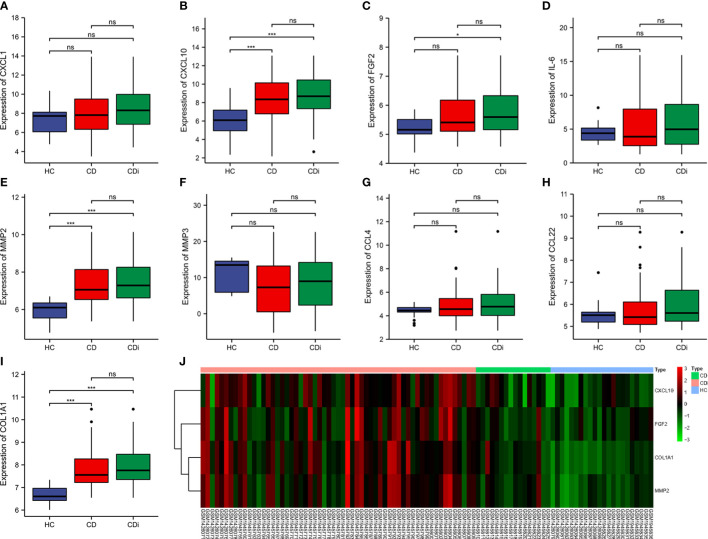
Validation of hub genes. The expression of 9 hub genes were validated by dataset GSE75214. The expression levels of COL1A1, CXCL10, and MMP2 were significantly higher in CD samples and active CD samples than in HC (*p* < 0.01) and the FGF2 was elevated in active CD compared to HC (*p*< 0.1) **(A–I)**. The expression levels of COL1A1, CXCL10, MMP2 and FGF2 were presented as a heat map **(J)**. *p< 0. 1, ***p< 0.01, ns, no significant difference.

To find reliable hub genes, these nine differential genes were re-validated using the dataset GSE75214. Among them, COL1A1, CXCL10, and MMP2 were expressed at significantly higher levels in both CD samples and active CD samples than in HC (*p*<0.01). FGF2 expression levels were elevated in active CD compared to HC (*p*<0.05), but not significantly different in CD (*p*>0.05) 0(**Figures 7A–I**). Ultimately, COL1A1, CXCL10, MMP2 and FGF2 were used as the hub genes in CD. The differences in the expression of hub genes in CD, active CD and HC were visualized by heat map (**Figure 7J**).

### Screening biomarkers for CD

**Figure 8:**
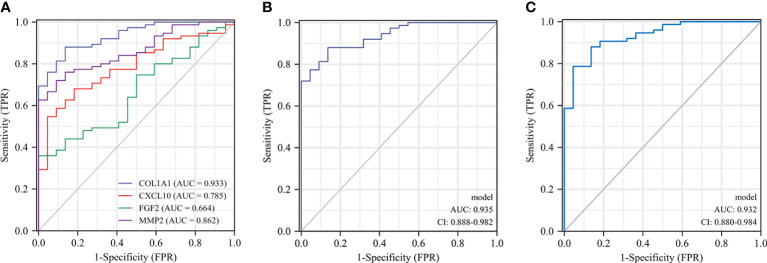
ROC curves of the 4 specifically expressed hub genes. Diagnostic value of COL1A1、CXCL10、MMP2 and FGF2 in CD using non-inflammatory tissues as controls by dataset GSE75214 **(A)**. Diagnostic value of COL1A1 and CXCL10 in CD (GSE75214 dataset) **(B)**. Diagnostic value of COL1A1 and MMP2 in CD (GSE75214 dataset) **(C)**.

The diagnostic validity of COL1A1, CXCL10, MMP2, and FGF2 for CD was verified by ROC analysis using the dataset GSE75214. the closer the AUC to 1, the better the diagnosis. the AUC was accurate at 0.7-0.9, and the AUC above 0.9 had a high specificity and sensitivity. In predicting HC and CD outcomes, there was high accuracy in the predictive power of COL1A1 (AUC = 0.933, CI = 0.885-0.981), MMP2 (AUC = 0.862, CI = 0.790-0.935), CXCL10 (AUC = 0.785, CI = 0.690- 0.881), and the predictive power of FGF2 had lower accuracy (AUC = 0.664, CI = 0.546-0.781) (**Figure 8A**). Combining some genes in the analysis, the combined AUC of COL1A1+MMP2 = 0.932 (CI = 0.880-0.984) and the combined AUC of COL1A1+CXCL10 = 0.935 (CI = 0.888-0.982) (**Figures 8B, C**). Thus, COL1A1 had the highest specificity and sensitivity among the individual indices, and COL1A1+CXCL10 had the best specificity among the combination of the two indices.

### Correlation analysis between hub genes and immune cells

**Figure 9:**
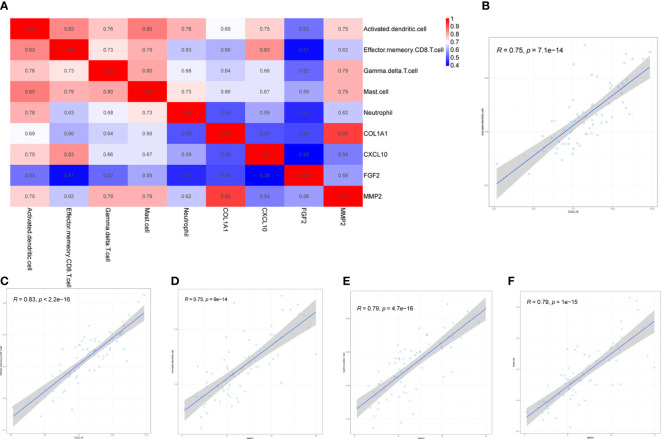
Correlation between hub genes and differential immune cells in CD. Correlation of 4 hub genes and 5 significantly differential immune cells **(A)**. CXCL10 was positively correlated with both activated dendritic cells and effector memory CD8^+^ T cells **(B, C)**. MMP2 was positively correlated with activated dendritic cells, gamma delta T cells and mast cells **(D–F)**.

Four biomarkers (COL1A1, CXCL10, MMP2, FGF2) were correlated with five immune cells with significant differences (effector memory CD8^+^ T cell, neutrophil, mast cell, activated dendritic cell, gamma delta T cell) derived by Wilcoxon test and LASSO regression analysis Correlation analysis was performed between (**Figure 9A**). Immune cells and biomarkers with significant correlation were screened by R≥0.75, *p*<0.001. The results showed that CXCL10 was positively correlated with activated dendritic cells (R=0.75, *p*=7.1e-14) and effector memory CD8^+^ T cells (R=0.83, *p*<2, 2e-16) (**Figures 9B, C**). MMP2 was positively correlated with activated dendritic cells (R=0.75, *p*=9e-14), γδ T cells (R= 0.79, *p*=4.7e-16), and mast cell (R=0.79, *p*=1e-15) were positively correlated (**Figures 9D–F**).

### Examination of biomarkers in animal models of CD fibrosis

**Figure 10:**
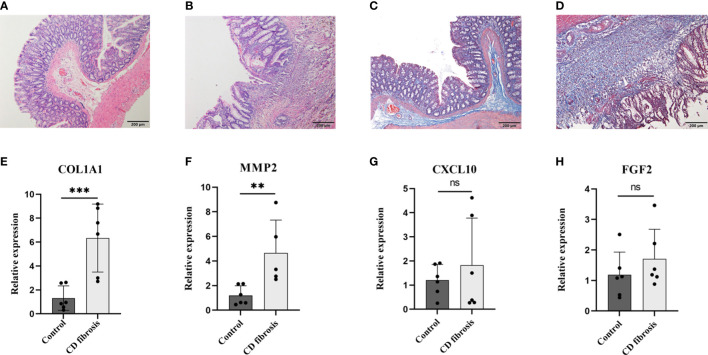
Examination of biomarkers in CD fibrosis rats. Representative HE stained and Masson stained images in normal **(A, C)** and model groups **(B, D)**, scale bar = 200µm. Statistical analysis of the expression levels of COL1A1、MMP2、CXCL10 and FGF2 in the control and model groups based on the RT-PCR assay **(E–H)**. Control: blank control rats (n=6); CD fibrosis, Crohn’s disease fibrosis model group rats (n=6). Data are presented as the mean ± SD. **p< 0. 05, ***p< 0.01, ns, no significant difference.

After verifying COL1A1, CXCL10, MMP2 and FGF2 in samples from CD patients, to further verify the expression of these four genes in Crohn’s disease fibrosis, we prepared a rat model of CD fibrosis using TNBS (17). HE staining showed that the normal group of rats had clear structure in all layers of colonic tissue, with intact mucosal morphology and regular arrangement, without ulceration and hyperplasia. In the model group, the colon tissue of rats was infiltrated by a large number of inflammatory cells, with disorganized tissue structure, destruction or deformation of glands and formation of cleft-like ulcers (**Figures 10A, B**). Masson staining showed that the mucosal layer and submucosal layer of the blank control group had a small amount of collagen fiber expression and neat arrangement, while the mucosal layer, submucosal layer and muscle layer of the CD fibrosis group had a large amount of blue collagen fiber expression and disorderly arrangement (**Figures 10C, D**). The PCR results showed that compared with the normal group COL1A1 (1.32 ± 1.02 vs 6.33 ± 2.84), CXCL10 (1.21 ± 0.65 vs 1.82 ± 1.95), MMP2 (1.21 ± 0.78 vs 4.66 ± 2.67) and FGF2 (1.18 ± 0.75 vs 1.71 ± 1.00), the results showed that COL1A1 and MMP2 expression were both significantly upregulated in Crohn’s disease intestinal fibrosis (*p*< 0.01) (**Figures 10E–H**).

## Discussion

Crohn’s disease is a disease of the autoimmune system characterized by recurrent episodes of intestinal inflammation. Its pathogenesis is thought to be closely related to changes in the type and number of immune cells, which disrupts the original immune homeostasis and thus induces an inflammatory cascade that leads to persistent damage to the intestinal mucosa (18). The results of immune infiltration and pathway enrichment also show that differential genes are most closely related to the immune response and its associated pathways. In recent years, the mainstay of CD therapy, biologics, also works by targeting immune cells or inflammatory cytokines to suppress the inflammatory response. However, patients on long-term biologic agents are at risk of secondary failure, with studies reporting primary nonresponse in more than one-third of patients on IFX and secondary nonresponse in 50% of those who do respond within one year (19). Therefore, it is particularly important to find new and more targeted immune cell types and immune-related molecular markers. In the present study, we explored the heterogeneity of immune cells between CD and normal tissues and identified validated biomarkers of CD by a comprehensive, objective and effective bioinformatics approach.

There was a significant heterogeneity of effector memory CD8^+^ T cells, neutrophils, mast cells, activated dendritic cells, and γδ T cells in the intestinal tissues of CD patients compared to normal tissues. It was found that effector memory CD8^+^ T cells, unlike other types of CD8^+^ T cells, could circulate throughout the body with the blood (20). Activity was significantly enhanced in the colon of active pediatric Crohn’s disease patients (21). In CD there was a significant increase in the number of γδ T in the intestine and circulation, and γδ T could recognize antigens directly without the need for antigen-presenting cells or MHC molecules (22). It could promote dendritic cell maturation by secreting Th1-type cytokines (IFN-γ, TNF-α) to activate the immune response (23, 24). Dendritic cells (DCs) DCs maintained low activity in normal human intestinal mucosa, however, the marker of DC activation, CD40, was significantly elevated in inflammatory active intestinal tissues of CD patients, and the expression of TLR-2 and TLR-4 was significantly upregulated (25).And DCs exhibited migration towards inflammatory tissues (26). Mast cells (MC) could maintain homeostasis in the organism through barrier defense, and the activity of MC was significantly enhanced in CD. Previous studies had found increased histamine levels and histamine 4 receptor (H4R) expression in CD patients, and histamine could exacerbate inflammation while promoting neutrophil recruitment and further inflammatory responses (8, 27). Neutrophils caused damage to the intestinal epithelial barrier by producing high levels of reactive oxygen species (ROS), and also produced leukotrienes, several pro-inflammatory cytokines such as TNF-α and IL-8, and neutrophil degranulation to recruit and activate more immune cells to exacerbate inflammation (28–30). All of the above are consistent with our findings of immune infiltration.

After locking in 9 differentially expressed genes, the reliability of the dataset GSE75214 was re validated, and finally four genes, COL1A1, CXCL10, MMP2, and FGF2, were identified as hub genes for CD. FGF2 was found to promote an increase in the number of colonic mucosal lymphatics in CD patients, thereby exacerbating the inflammatory response (31). MMP2 expression levels in CD were significantly increased in intestinal epithelial and infiltrating immune cells, which may exacerbate the disease by increasing intestinal barrier dysfunction through increased FITC dextrose translocation (32–34). ROC regression analysis showed that the combination of CXCL10 and COL1A1 had the best sensitivity and specificity in diagnosing CD. CXCL10, an inflammatory chemokine that could be induced by IFN-γ, was produced by neutrophils, fibroblasts, and dendritic cells, homing leukocytes to inflamed tissues and causing the persistence of inflammation (35, 36), and played a role in the pathogenesis of several autoimmune system diseases (37). COL1A1 was significantly elevated in the intestinal fibrosis phenotype (38, 39).

Given the close relationship between immune infiltrating cells and hub genes in CD, we analyzed the correlation between 5 types of immune cells with significant differences and 4 hub genes. The results indicate that CXCL10 is positively correlated with activated dendritic cells and effector memory CD8^+^T cells. MMP2 is positively correlated with activated dendritic cells, γδ T cells, and mast cells. Previous studies had shown that NOD2 and IFN-γ could synergistically induce DCs to elevate CXCL10 expression and thus chemotactic CXCR3-dependent small intestinal CD8^+^ T cell migration during T cell activation leads to an increased intestinal inflammatory environment (40–42).MMP2 could directly activate DCs to secrete a variety of inflammatory cytokines and also acted as a NF-κB dependent gene regulating NF-κB in bone marrow-derived mast cells (43). A correlation between MMP2 and elevated γδ T cells had also been observed in other inflammation-related diseases (44). In conclusion, MMP2 and CXCL10 may pass through DC cells, mast cell, γδ T cells and effector memory CD8^+^T cells regulate innate and adaptive immune responses, thereby exacerbating intestinal inflammatory response.

More than one-third of clinical CD patients would experience recurrent intestinal obstruction due to chronic transmural inflammation and recurrent ulcer repair with excessive extracellular matrix (ECM) deposition forming scar tissue, resulting in fibrotic stenosis of the intestinal lumen and eventual surgical resection of the diseased bowel (45, 46). However, due to the relatively long course of fibrosis that progressed to the stage of intestinal luminal stricture, there was a lack of clinical screening modalities that could identify it early. Therefore, it was particularly important to find molecular markers targeting CD intestinal fibrosis for early diagnosis. Based on this, this study identified molecular markers of Crohn’s disease and used TNBS induced Crohn’s disease intestinal fibrosis rat model for experimental validation. The results showed that COL1A1 and MMP2 were significantly elevated in the colon tissue of CD induced intestinal fibrosis rats. The pathological process of fibrosis mainly involves excessive deposition of ECM, with downregulation of matrix metalloproteinases (MMPs) and upregulation of tissue inhibitors of metalloproteinases (TIMPs) leading to a disruption of the balance between extracellular matrix deposition and degradation as typical manifestations. MMP2 belongs to MMPs and can inhibit the degradation of type I collagen; COL1A1 exacerbates the ECM process, so both can exacerbate the process of intestinal fibrosis (47–49). The results of immune infiltration showed a positive correlation between MMP2 and activated dendritic cells, gamma delta T cells, and mast cells. The experimental study found that the infiltration of mast cell in the fibrotic intestinal tissue increased significantly (50), γδ T cell receptor positive cells participate in the pathological process of liver fibrosis (51). Based on the results of this experiment, COL1A1 and MMP2 may participate in the pathological process of CD intestinal fibrosis by regulating immune responses and ECM processes. In the future, COL1A1 and MMP2 may serve as molecular markers of CD intestinal fibrosis.

## Conclusion

This study not only analyzed the biomarkers of CD, but also established validated biomarkers closely related to CD immune response through macroscopic immune cell infiltration analysis, making the research results more reliable. In addition, this study further validated the genes related to intestinal fibrosis in the hub gene through *in vivo* experiments, providing data support for the early diagnosis of intestinal fibrosis. However, there are certain shortcomings in current research. Firstly, the clinical details corresponding to the research data are not complete enough. This study did not validate and conduct in-depth analysis of miRNAs processed by integrating multiple databases.

## Data availability statement

The datasets presented in this study can be found in online repositories. The names of the repository/repositories and accession number(s) can be found in the article/supplementary material.

## Ethics statement

The animal study was reviewed and approved by Ethics Committee of Experimental Animal Center, Yueyang Hospital of Integrated Traditional Chinese and Western Medicine, Shanghai.

## Author contributions

RH, HW, and HL conceived the study. RH, WW, ZC, and JC obtained reagents and performed experiments. RH, QQ, HZ, and BC analyzed the data and wrote the manuscript. All authors contributed to the article and approved the submitted version.
